# Positive Anti-SSA/Ro Antibody in a Woman with SARS-CoV-2 Infection Using Immunophenotyping: A Case Report

**DOI:** 10.3390/medicina56100521

**Published:** 2020-10-05

**Authors:** Po-I Huang, Ting-Chun Lin, Feng-Cheng Liu, Yi-Jung Ho, Jeng-Wei Lu, Te-Yu Lin

**Affiliations:** 1 School of Medicine, National Defense Medical Center, Taipei 114, Taiwan; ian111924@gmail.com; 2 School of Pharmacy, National Defense Medical Center, Taipei 114, Taiwan; d0938138876@gmail.com; 3 Rheumatology/Immunology and Allergy, Department of Internal Medicine, Tri-Service General Hospital, National Defense Medical Center, Taipei 114, Taiwan; lfc10399@yahoo.com.tw; 4 Graduate Institute of Life Science, National Defense Medical Center, Taipei 114, Taiwan; ejung330@gmail.com; 5 Department of Biological Sciences, National University of Singapore, Singapore 117543, Singapore; jengweilu@gmail.com; 6 Division of Infectious Diseases and Tropical Medicine, Department of Internal Medicine, Tri-Service General Hospital, National Defense Medical Center, Taipei 114, Taiwan

**Keywords:** COVID-19, SARS-CoV-2, Sjögren’s syndrome, autoimmune, immunophenotyping

## Abstract

The clinical spectrum of novel coronavirus infection appears to be wide, encompassing asymptomatic infection, mild upper respiratory tract illness, and severe viral pneumonia, with respiratory failure and even death. Autoantibodies, especially antiphospholipid antibodies, can occur in severe infections. Other autoantibodies are seldom reported. Here, a 60-year-old female patient without dry-mouth symptoms detected positive for anti-60 kDa SSA/Ro antibodies on day 43 after severe acute respiratory syndrome coronavirus 2 (SARS-CoV-2) infection. To investigate this unique clinical case of SARS-CoV-2 infection, immunological characteristics of this case were detected by using flow cytometry and were compared to the other three groups of patients—health subjects, 2019 novel coronavirus disease (COVID-19) recovery patients, and Sjögren’s syndrome (SS) patients. Monitoring the autoantibody level and the development of subsequently related autoimmune diseases are warranted after SARS-CoV-2 infection.

## 1. Introduction

Severe acute respiratory syndrome coronavirus 2 (SARS-CoV-2) infection is spreading at an alarming rate worldwide. Clinical manifestations include fever, cough, shortness of breath, sore throat, myalgia, arthralgia, headache, rhinorrhea, nausea, vomiting, diarrhea, and olfactory and gustatory dysfunctions. Autoantibodies (particularly antiphospholipid antibodies) are commonly encountered in cases of severe infection [1]. In autoimmune diseases, anti-SSA/Ro antibodies are the most common autoantibodies whose pathological role are still controversial. Anti-SSA/Ro antibodies were frequently observed in association with Sjögren’s syndrome (SS), systemic lupus erythematosus (SLE), rheumatoid arthritis (RA), subacute cutaneous lupus erythematosus (SCLE), neonatal lupus erythematosus (NLE), systemic sclerosis (SSc), polymyositis (PM), dermatomyositis (DM), primary biliary cirrhosis (PBC), and type 1 autoimmune hepatitis (AIH-1). In particular, the incidence of anti-SSA/Ro positive was 70–100% in SS patients and 40–90% in SLE patients [2]. Autoimmune characteristics exist at the recovery stage of some patients with severe and critical cases of the new 2019 novel coronavirus disease (COVID-19). Among twenty COVID-19 subjects, the prevalence of anti-52 kDa or anti-60 kDa SSA/Ro antibodies were 20% and 25%, respectively [3]. SARS-CoV-2 infection also had correlation with rheumatic disease [4]. However, other autoantibodies are seldom reported. In this case report, we described a female patient without dry-mouth symptom who developed positive anti-60 kDa SSA/Ro antibody at 43 days after SARS-CoV-2 infection. For this patient, we reviewed the previous medical history of this case in the hospital and conducted patient consultation. Therefore, we further ruled out the possibility of other autoimmune diseases. Comparisons of immunological characteristics between patients with this case, health subjects, COVID-19 recovery patients and SS patients were conducted to discuss this unique clinical presentation of SARS-CoV-2 infection. 

## 2. Case Report

**Figure 1:**
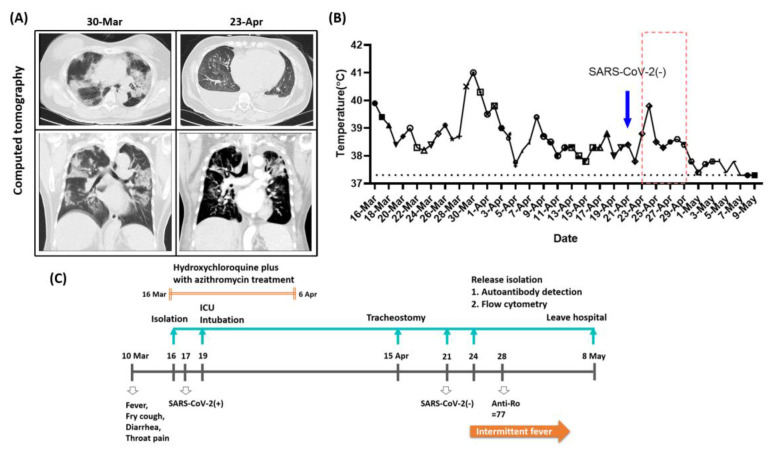
The progress of the disease and the course of clinical treatment. (**A**) Computed tomography (CT) of the chest. The cross-section and longitudinal-section of CT revealed diffuse ground-glass opacities throughout both lungs on 17 March 2020, and recovered on 23 April 2020. (**B**) The temperature record during the whole hospitalized period. The blue arrow means severe acute respiratory syndrome coronavirus 2 (SARS-CoV-2) (-). The red dotted frame means a period of intermittent fever. (**C**) The disease progression of the case. The gray line means the total disease progression of the case. The hollow arrow points out the time points from the beginning symptoms, SARS-CoV-2 positive to negative, and the level of anti-Ro. The yellow arrow means a period of intermittent fever. The green line means the hospitalization until discharge. The green arrow means the medical treatments of the case.

A 60-year-old female had a history of hypertensive cardiovascular disease with regular follow up. The case departed Taiwan on 2 January 2020, for a trip to the Czech Republic and New York. On 10 March 2020, she presented with fever, dry cough, diarrhea, and throat pain. The case returned to Taiwan on 16 March 2020, at which time she was still suffering from coughing. Upon arrival at our emergency department, she was admitted to the isolation ward. A physical examination performed on admission showed bilateral crackles. The initial laboratory results were as follows: (1) white cell count, 4.04 × 10^3^ cell/mm^3^; (2) absolute neutrophil count, 3.06 × 10^3^ cell/mm^3^; (3) absolute lymphocyte count, 0.69 × 10^3^ cell/mm^3^; (4) hemoglobin levels, 13.4 g/dL; (5) platelets, 149 × 10^3^ cell/mm^3^; (6) Ferritin, 707.9 μg/L; (7) C-reactive protein, 5.77 mg/dL. Chest radiographs showed increased infiltration over bilateral lower lung fields (Figure 1A). Chest computed tomography scans revealed diffuse ground-glass opacities throughout both lungs. Throat swabs obtained on 17 March 2020, tested positive for SARS-CoV-2. On 19 March 2020, the case was transferred to the intensive care unit on account of respiratory failure. Endotracheal insertion was performed until 15 April 2020, at which time tracheostomy was done due to difficulties in weaning from the ventilator. Besides, the patient received hydroxychloroquine plus with azithromycin treatment from 16 March to 6 April. According to Taiwan Centers for Disease Control (CDC) regulations, three consecutive samples of throat swab returned negative results for SARS-CoV-2, and the case does not require isolation measures. Although the results were negative for SARS-CoV-2, the case still continued to suffer from intermittent fever. (Figure 1B). Following a complete fever workup, the autoimmune disease survey was performed, the case presented anti-60 kDa SSA/Ro antibodies, which is an important criterion for diagnosis with SS, an autoimmune disease. The clinical condition improved gradually under supporting care, and the case recovered well and was discharged on 18 May 2020 (Figure 1C). This study was approved by the Institutional Review Board (IRB) of Tri-Service General Hospital, National Defense Medical Center, Taipei, Taiwan, and complied with guidelines (IRB No. 2-106-05-001, date of approval 26 January 2020; Patient informed consent No. 2-106-05-001.

Comparative analysis of the case was conducted versus a group of patients who had recovered from SARS-CoV-2 infection without positive anti-60 kDa SSA/Ro antibody (recovery group, *n* = 4), patients diagnosed with SS (*n* = 4), and healthy volunteer (healthy control group, *n* = 4). For the recovery group, there are two males, aged 22 and 66 years old; and two females, aged 60 and 28 years old, who had mild symptoms of COVID-19, and who have received hydroxychloroquine plus with azithromycin treatment, respectively. The above voluntary health control and patient groups are all from Tri-Service General Hospital and receive treatment. The results of blood biochemistry analysis and autoantibody tests were collected from the medical records of Tri-Service General Hospital. Furthermore, immunophenotyping of whole peripheral blood by flow cytometry was used to identify potential regulators of immune cells. The immune cells were analyzed using a Beckman Coulter, CytoFLEX. Briefly, whole blood samples were added separately into three kits—DuraClone IM T cell subsets Tube (Beckman Coulter, B53328), DuraClone IM Treg Tube (Beckman Coulter, B53346) and DuraClone IM B cell Tube (BECKMAN COULTER, B53318). Anti-human CD95-BV605 (BioLegend, 305628), anti-human CD366-BV650 (BioLegend, 345028), anti-mouse/human killer-cell lectin like receptor G1 (KLRG1)-BV785 (BioLegend, 138429), anti-human HLA-DR-BV650 (BioLegend, 307650), anti-human CD62L (BioLegend, 304834)-BV605, anti-human CD127-BV785 (BioLegend, 351330), CD8-APC-Alexa Fluor 700 (Beckman Coulter, A66332) were added to kits in advance. The detailed setting and the cells gating are listed in the Supplementary Table S1 and Figures. The analysis was performed in strict adherence to the protocols outlined by the manufacturer. The raw data obtained from flow cytometry were analyzed using Kaluza 2.1.1 software. Comparisons of the case and other groups were based on mean ± 3SD (Table 1). The data of the case that was higher or lower than the compared group are respectively indicated using ↑ or ↓.

## 3. Discussion

Current research shows that spike protein of SARS-CoV-2 can bind to angiotensin, converting enzyme 2 (ACE2) receptor on the host cells and further cause inflammatory reactions in related organs [5]. The ACE2 receptor can be found in the salivary gland and is one of the targets of SARS-CoV-2 [6]. After virus enters into host cells, the interaction of virus and cells stimulates the organism to produce neutralizing antibodies and to mediate the cellular immune response. SARS-CoV-2 infection may contribute to serious and critical illness and mortality through autoimmunity [7]. Anti-SSA/Ro antibodies were autoantibodies that presented in some autoimmune diseases [2]. Our case report shows that the antibody levels of anti-60 kDa SSA/Ro, anti-La, anti-Histone, and connective tissue were higher in the case than in the recovery group; however, only the anti-60 kDa SSA/Ro levels were clinically significant. In hematology tests, the platelet and neutrophil counts were higher in the case than in the recovery group, whereas the numbers of lymphocytes and basophils were lower. Note that after recovering from severe COVID-19, the case had a high neutrophil-to-lymphocyte (N-L) ratio, which has previously been identified as an independent risk factor for mortality in hospital [8].

**Figure 2:**
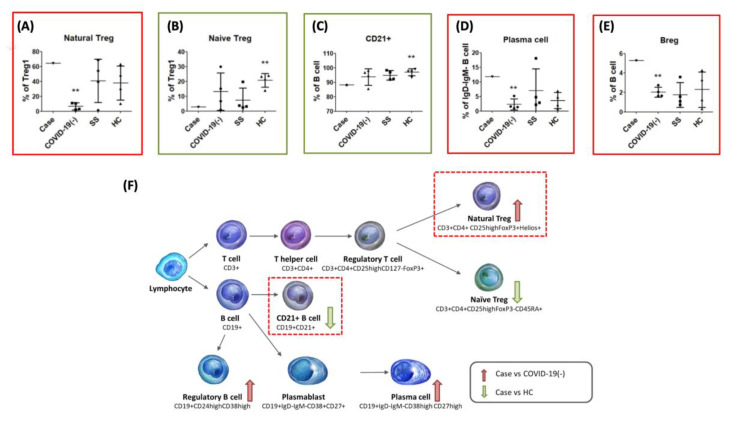
Immunophenotyping analysis of whole peripheral blood of the case by flow cytometry. (**A**,**D**,**E**). The case revealed the significant increase of natural Treg, Breg, and plasma cells compared with COVID-19 (-), but the significant decrease of (**B**,**C**) naive Treg and CD21+ B cells compared with healthy control. (**F**) The immunophenotyping conclusion of the case. Abbreviation: healthy control (HC) and Sjögren’s syndrome (SS). Statistical significance was determined via a t-test compared with COVID-19 (-) or healthy control: * *p* < 0.05; and ** *p* < 0.05, respectively.

Immunophenotyping of whole blood demonstrated that the ratio of programmed cell death protein 1 (PD-1) + of T helper cells, KLRG1 + of effector T helper cells, and Tim-3 + of central memory cytotoxic T cells was significantly higher in the case than in the recovery group. Besides, KLRG1 expression of the case decreased in some T cell subgroups, such as effector T cells or effector memory T cells than healthy control. Note that these biomarkers are negative regulators of T cells, and the elevated expression of PD-1 has been associated with the functional failure of T helper cells. PD-1 inhibited T cell recruitment into the follicle. PD-1 further limited the upregulation of C-X-C motif chemokine receptor 3 (CXCR3) in follicular T helper cells, where the PD-1 and/or PD-L1 interaction between a single T helper and B cells optimizes B cell competition and affinity maturation [9]. The co-inhibitory receptor killer-cell lectin like receptor G1 (KLRG1) is expressed on NK cells and antigen-experienced T cells and has been characterized as a senescent biomarker on the effector T cells. The KLRG1 emphasizes the functional defects that appear in the process of T cell differentiation with age. This defect may be actively maintained, in part through inhibitory receptor signaling [10]. T cell immunoglobulin mucin-3 (Tim-3) has been implicated in the mediation of T cell exhaustion or dysfunction related to the restriction of long-lived memory T cells. However, Tim-3 is more similar to costimulatory receptors that are up-regulated after T cells activation, rather than a dominant inhibitory protein like PD-1 [11]. Thus, the elevated expression of these biomarkers might be an indication of an exhausted immune system due to the rigors of fighting off a severe disease. Some biomarkers, such as KLRG1, should be followed to figure their role in severe COVID-19. We did not observe a significant difference between the case and recovery group in terms of the overall percentage of Treg cells; however, the case had a higher percentage of natural Tregs (CD4 + CD25highFoxP3 + Helios +) (Figure 2A) and lower percentage of naive Tregs (CD3 + CD4 + CD25highFoxP3 − CD45RA +) (Figure 2B). Liu et al. have reported elevated levels of circulating CD4 + Helios + FoxP3+ cells in patients with primary diagnosis of SS, which might contribute to suppressing autoimmunity [12]. The immunological analysis of our case implies that monitoring subsequent development of SS is warranted.

Saadoun et al. observed high frequencies of autoreactive and unresponsive CD21 − /low B cell populations that associated with lymphoproliferation in patients with primary of SS [13]. Glauzy et al. also demonstrated that the frequency of CD21 − /low B cells was increased in the peripheral blood of 5 of 8 SS patients [14]. In our case, the ratio of CD21+ marginal zone B cells and switch memory B cells as well as PD-1+ of marginal cells and HLADR+ of switch memory B cells was far lower in the case than in the recovery group (Figure 2C). Note that there was no significant difference between the case and recovery group in terms of the percentage of plasmablasts; however, the case presented a relatively high percentage of plasma cells (Figure 2D) and Breg cells (Figure 2E) compared to the recovery group (Table 1). This was an indication that the immune system of the case was activated, under which conditions the body would be expected to produce a larger number of antibodies, thereby increasing the likelihood of developing an autoimmune disease, such as SS. 

However, our evidence shows that this case had a more similar trend to SS using immunophenotype analysis (Figure 2F). The scenario of anti-60 kDa SSA/Ro positive antibody in this case is the destruction of the epithelium of the exocrine glands, as a consequence of abnormal B cell responses to the autoantigens Ro/SSA. Monitoring sicca symptoms and subsequent SS development are warranted after SARS-CoV-2 infection. In addition, due to limited samples, the current evidence is still preliminary, and the number of patients and further verification experiments will be expanded in the future.

## 4. Conclusions

In conclusion, this is the first report on the immunophenotyping of a COVID-19 case with elevated autoantibodies. This case report may provide useful suggestions for rheumatologists or infection specialists in predicting prognosis, monitoring therapeutic responses, and even choosing more appropriate therapeutic regimens for COVID-19-infected patients. Moreover, further studies in larger cohorts of COVID-19-infected patients are advocated to clarify this opinion.

## Figures and Tables

**Table 1:** The Clinical Characteristics and Flow Cytometry Analysis of Different Groups.

		The Case	Mean ± 3SD		
Groups			Recovered COVID-19 (*n* = 4)	Sjögren’s Syndrome (*n* = 4)	Healthy Control (*n* = 4)
**Clinical Characteristics**					
	Age (years)	60	45.25 ± 70.54	56.75 ± 20.11	46.75 ± 16.68
	Duration	43 days	41.75 ± 49.56 days	3.00 ± 8.49 years	-
	Severity	Severe	Mild	-	-
	ESSDAI	-	-	2.00 ± 4.24	-
Autoantibody	Anti-Ro (U/mL)	77	<0.3	76.00 ± 106.24	-
	Anti-La (U/mL)	3.7	<0.3	5.70 ± 5.94	-
	Anti-Histone (U/mL)	1.2	-	-	-
	Connective Tissue (Ratio)	1.4	-	3.98 ± 7.90	-
Hematology Test	WBC (*10^3^/mm^3^)	8.43	6.64 ± 6.54	7.52 ± 8.76	-
	RBC (*10^6^/mm^3^)	3.5	4.55 ± 1.31	4.32 ± 2.12	-
	Hb (g/dL)	10.7	13.35 ± 2.74	12.20 ± 4.00	-
	PLT (*10^3^/mm^3^)	367	220.25 ± 25.14 (↓)	284.00 ± 263.49	-
	Neutrophil (%)	81.7	54.53 ± 12.80 (↓)	66.53 ± 31.74	-
	Lymphocyte (%)	13.6	33.38 ± 19.32 (↑)	26.68 ± 25.12	-
	Neutrophil/Lymphocyte (Ratio)	6.01	1.69 ± 1.26 (↓)	2.77 ± 3.54	-
	Basophil (%)	0.2	0.90 ± 0.65 (↑)	0.53 ± 0.62	-
Biochemistry Test	BUN (mg/dL)	16	11.25 ± 3.77 (↓)	17.25 ± 33.26	-
	ALT (IU/L)	34	11.50 ± 16.34 (↓)	11.75 ± 5.12 (↓)	-
	CRP (mg/dL)	1.57	<0.1 (↓)	0.07 ± 0.41 (↓)	-
**T Cell Panel**					
Lymphocytes/Leukocytes		34.15	40.26 ± 38.92	35.17 ± 7.28	43.88 ± 60.99
T cells (CD3 +)		74.26	70.92 ± 18.94	70.06 ± 21.43	65.50 ± 13.07
T helper cells (CD3 + CD4 +)		65.26	51.27 ± 33.59	66.16 ± 33.16	62.24 ± 37.59
KLRG1		15.59	26.05 ± 26.17 ^#^	27.44 ± 21.86 ^#^	70.80 ± 97.94
PD-1		47.35	34.44 ± 5.50 (↓)	34.61 ± 37.35	32.99 ± 27.36
Tim-3		0.86	0.59 ± 0.79	1.33 ± 2.14	14.42 ± 59.75
Naïve T helper cells (CD3 + CD4 + CCR7 + CD45RA +)		27.25	27.00 ± 44.66	31.84 ± 43.62	39.64 ± 36.43
KLRG1		2.45	15.73 ± 27.05	20.91 ± 29.42	58.22 ± 144.22
PD-1		3.02	3.29 ± 3.29	2.88 ± 3.51	2.84 ± 6.03
Tim-3		0.32	0.16 ± 0.30	0.33 ± 0.61	4.86 ± 27.00
Central memory T helper cells (CD3 + CD4 + CCR7 + CD45RA -)		55.18	54.39 ± 41.55	54.45 ± 49.40	48.07 ± 30.10
KLRG1		16.22	26.16 ± 33.05 ^#^	30.88 ± 31.80 ^#^	74.65 ± 85.37
PD-1		59.23	41.93 ± 27.52	43.68 ± 43.09	47.70 ± 19.18
Tim-3		1.06	0.65 ± 1.04	1.47 ± 2.38	18.17 ± 65.66
Effector T helper cells (CD3 + CD4 + CCR7 - CD45RA +)		1.1	0.36 ± 1.52	0.13 ± 0.26 (↓)	0.32 ± 1.03
KLRG1		75.68	52.82 ± 16.16 (↓) ^#^	40.79 ± 34.61 (↓) ^#^	91.98 ± 9.14 (↑)
PD-1		80.89	72.71 ± 18.79	57.81 ± 49.83	65.86 ± 58.13
Tim-3		1.49	0.62 ± 2.33	5.34 ± 10.04	13.52 ± 65.06
Effector memory T helper cells (CD3 + CD4 + CCR7 - CD45RA -)		16.47	18.26 ± 8.26 ^#^	13.59 ± 31.79	11.98 ± 14.40
KLRG1		31.2	40.95 ± 28.12 ^#^	38.46 ± 43.25 ^#^	92.02 ± 31.60 (↑)
PD-1		78.66	62.92 ± 31.40	68.31 ± 37.08	72.38 ± 49.70
Tim-3		1.05	1.32 ± 2.62	2.89 ± 5.37	22.61 ± 77.90
Cytotoxic T cells (CD3 + CD8 +)		25.46	38.80 ± 29.16	23.86 ± 40.24	27.77 ± 22.00
KLRG1		39.13	49.27 ± 30.53 ^#^	45.92 ± 51.78 ^#^	89.38 ± 41.50 (↑)
PD-1		57.34	37.36 ± 33.09	31.71 ± 36.92	34.84 ± 40.54
Tim-3		1.95	1.18 ± 1.38	1.79 ± 1.24	2.21 ± 4.65
Naïve cytotoxic T cells (CD3 + CD8 + CCR7 + CD45RA +)		27.72	21.43 ± 64.43	32.17 ± 49.83	35.40 ± 56.95
KLRG1		4.12	21.13 ± 20.39 ^#^	23.05 ± 60.99	66.49 ± 116.17
PD-1		5.03	9.03 ± 22.21	6.04 ± 8.28	7.70 ± 15.61
Tim-3		1.24	0.99 ± 3.87	0.88 ± 0.57 ^#^	0.57 ± 0.51 (↓)
Central memory cytotoxic T cells (CD3 + CD8 + CCR7 + CD45RA -)		10.89	12.76 ± 7.08	21.22 ± 37.55	14.82 ± 14.01
KLRG1		22.52	40.44 ± 18.67 ^#^	32.02 ± 32.84 ^#^	84.74 ± 64.38
PD-1		73.23	59.52 ± 29.93	45.59 ± 43.46	57.54 ± 36.95
Tim-3		2.38	1.04 ± 1.26 (↓)	2.21 ± 2.40	2.45 ± 4.24
Effector cytotoxic T cells (CD3 + CD8 + CCR7 - CD45RA +)		17.89	19.33 ± 66.97	14.46 ± 29.48	23.74 ± 50.89
KLRG1		63.89	53.79 ± 46.75 ^#^	68.41 ± 52.49 ^#^	98.67 ± 5.57 (↑)
PD-1		53.98	31.93 ± 65.59	22.37 ± 25.24 (↓)	37.15 ± 60.29
Tim-3		2.23	1.43 ± 1.61	1.95 ± 2.32	2.63 ± 4.22
Effector memory cytotoxic T cells (CD3 + CD8 + CCR7-CD45RA-)		43.5	46.49 ± 58.75	32.15 ± 54.01	26.05 ± 16.01 (↓)
KLRG1		55.4	66.67 ± 58.52 ^#^	67.55 ± 20.33 ^#^	95.66 ± 21.43 (↑)
PD-1		88.08	57.66 ± 83.82	53.18 ± 43.46	62.24 ± 57.13
Tim-3		2.19	1.76 ± 4.29	2.59 ± 2.95	3.46 ± 6.71
**Treg Cell Panel**					
T helper cells					
Treg (CD3 + CD4 + CD25high)		6.69	5.43 ± 6.08	4.93 ± 5.78	6.65 ± 4.84
Natural Treg (CD3 + CD4 + CD25highFoxP3 + Helios +)		64.38	6.75 ± 14.31 (↓)	40.85 ± 86.61	37.90 ± 68.42
Naïve Treg (CD3 + CD4 + CD25highFoxP3 - CD45RA+)		2.77	13.18 ± 37.88	7.27 ± 25.09 ^#^	20.66 ± 13.77 (↑)
**B Cell Panel**					
Lymphocytes					
B cells (CD19 +)		12.56	21.82 ± 64.71	6.48 ± 11.12	10.03 ± 8.87
CD21+		88.17	93.72 ± 17.09	94.83 ± 9.76	96.87 ± 7.29 (↑)
HLADR+		97.12	96.93 ± 3.18	97.02 ± 4.80	96.33 ± 12.09
PD-1+		11.75	47.43 ± 40.69	28.48 ± 43.97	30.01 ± 64.67
Marginal zone B cells (CD19 + CD27 + IgD +)		5.77	12.35 ± 16.70	5.80 ± 2.65 ^#^	15.23 ± 25.45
CD21+		89.36	96.93 ± 3.55 (↑)	92.79 ± 16.86	97.36 ± 5.42 (↑)
HLADR+		95.48	94.08 ± 13.34	97.64 ± 3.25	94.72 ± 15.88
PD-1+		16.75	57.88 ± 28.40 (↑)	34.02 ± 44.40	33.39 ± 65.69
Naïve B cells (CD19 + CD27 – IgD +)		69.94	52.70 ± 44.17	65.57 ± 9.63	55.80 ± 30.40
CD21+		96.81	96.74 ± 7.26	98.79 ± 2.41	98.58 ± 4.10
HLADR+		98.85	98.07 ± 3.25	97.91 ± 4.68	96.45 ± 12.25
PD-1+		11.18	52.19 ± 43.81	31.92 ± 47.65	32.11 ± 69.86
Switch memory B cells (CD19 + CD27 + IgD-)		18.23	24.55 ± 27.77	23.34 ± 7.77	21.95 ± 14.87
CD21 +		63.18	94.21 ± 14.69 (↑)	87.66 ± 20.81 (↑)	94.50 ± 13.18 (↑)
HLADR +		91.04	96.36 ± 5.02 (↑)	94.55 ± 7.09	96.68 ± 9.24
PD-1 +		11.63	38.79 ± 42.40	18.87 ± 34.43	24.27 ± 56.87
Double negative B cells (CD19 + CD27 – IgD -)		6.06	10.39 ± 23.53	5.30 ± 3.70	7.02 ± 8.30
CD21 +		62.59	77.54 ± 34.60	83.43 ± 33.56	88.69 ± 32.39
HLADR +		96.97	97.55 ± 4.52	96.47 ± 7.02	96.49 ± 17.37
PD-1 +		13.94	41.26 ± 35.18	22.78 ± 42.48	27.25 ± 75.70
Breg (CD19 + CD24highCD38high)		5.28	2.03 ± 1.55 (↓)	1.76 ± 3.83	2.29 ± 5.47
Plasmablasts (CD19 + IgD - IgM - CD38 + CD27 + /CD19 + CD27 + CD38 + B cells)		54.92	59.52 ± 23.35	68.25 ± 55.27	58.29 ± 57.36
Plasma cells (CD19 + IgD – IgM - CD38high CD27high/CD19 + IgD – IgM - B cells)		11.77	2.27 ± 5.76 (↓)	6.97 ± 22.53	3.58 ± 8.25

1. The means of ↑↓ presented each group’s mean ± 3* standard deviation is higher or lower than the case’s value. 2. (+) and (-) indicate positive and negative, respectively. 3. Mann–Whitney U tests were used for comparison of each groups with healthy control, ^#^ *p* < 0.05.

## Supplementary Materials

The following are available online at https://www.mdpi.com/1010-660X/56/10/521/s1.
