# Supplementary material for: Positive Anti-SSA/Ro Antibody in a Woman with SARS-CoV-2 Infection Using Immunophenotyping: A Case Report
Source: Medicina (Kaunas). 2020 Oct 5;56(10):521. doi: 10.3390/medicina56100521 (PMC7600347; doi:10.3390/medicina56100521)
Supplement: Supplementary file 1 [file medicina-56-00521-s001.pdf]

Supplementary Table 1

The antibodies of T cell, Treg and B cell panel corresponding to fluorescent dyes

Laser	Fluorescence dye	DuraClone IM T cell subsets Tube	DuraClone IM Treg Tube	DuraClone IM B cell Tube
**488nm**	**FITC**	CD45RA	CD45RA	IgD
	**PE**	CCR7	CD25	CD21
	**ECD**	CD28	-	CD19
	**PC5.5**	PD-1	CD39	PD-1
	**PC7**	CD27	CD4	CD27
**638nm**	**APC**	CD4	FoxP3	CD24
	**APC-A700**	CD8	CD8*	-
	**APC-A750**	CD3	CD3	CD38
**405nm**	**Pacific Blue (PB)**	CD57	Helios	IgM
	**Krome Orange (KrO)**	CD45	CD45	CD45
	**BV605**	CD95*	CD62L*	CD95*
	**BV650**	Tim-3*	HLADR*	HLADR*
	**BV780**	KLRG1*	CD127*	-

*The antibodies were extra-added in the DuraClone IM kits, each antibody was added 5ul in one reaction.

Supplementary Figure 1. The cell gating of T cell immunophenotyping

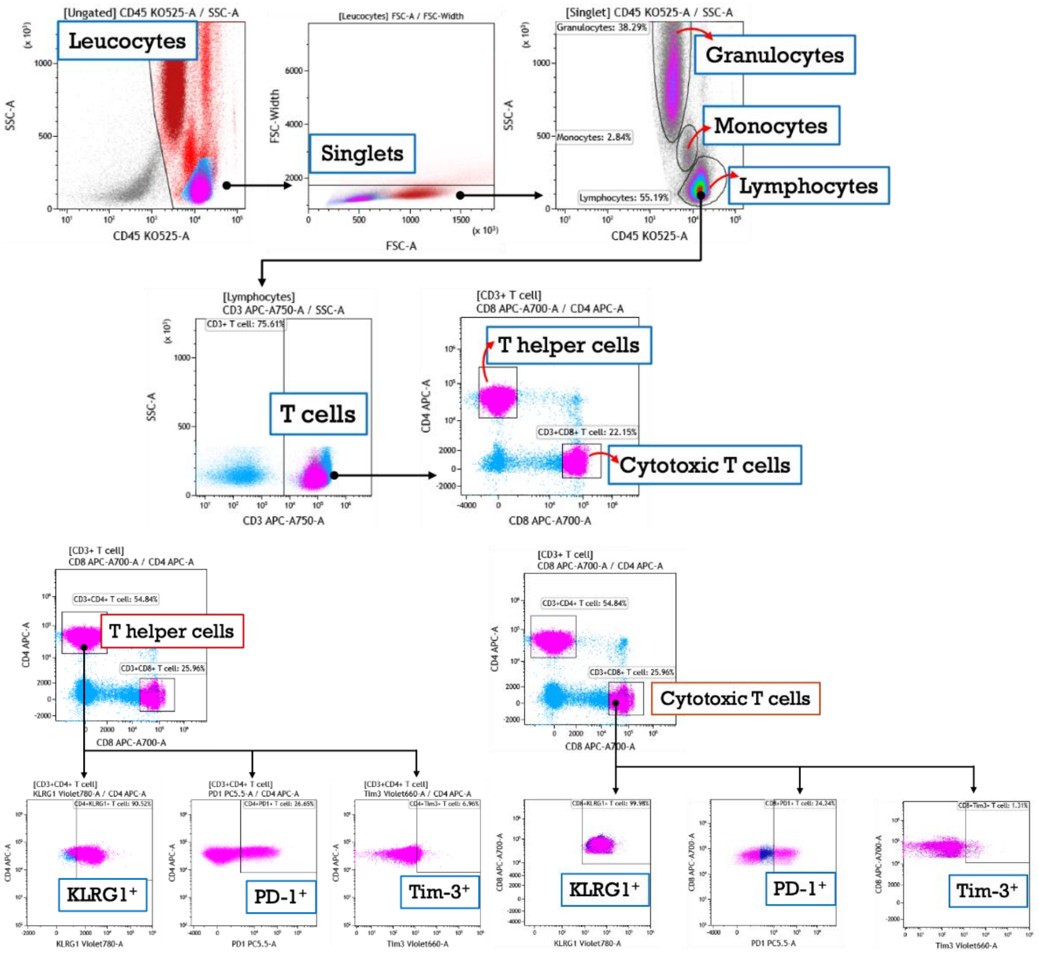

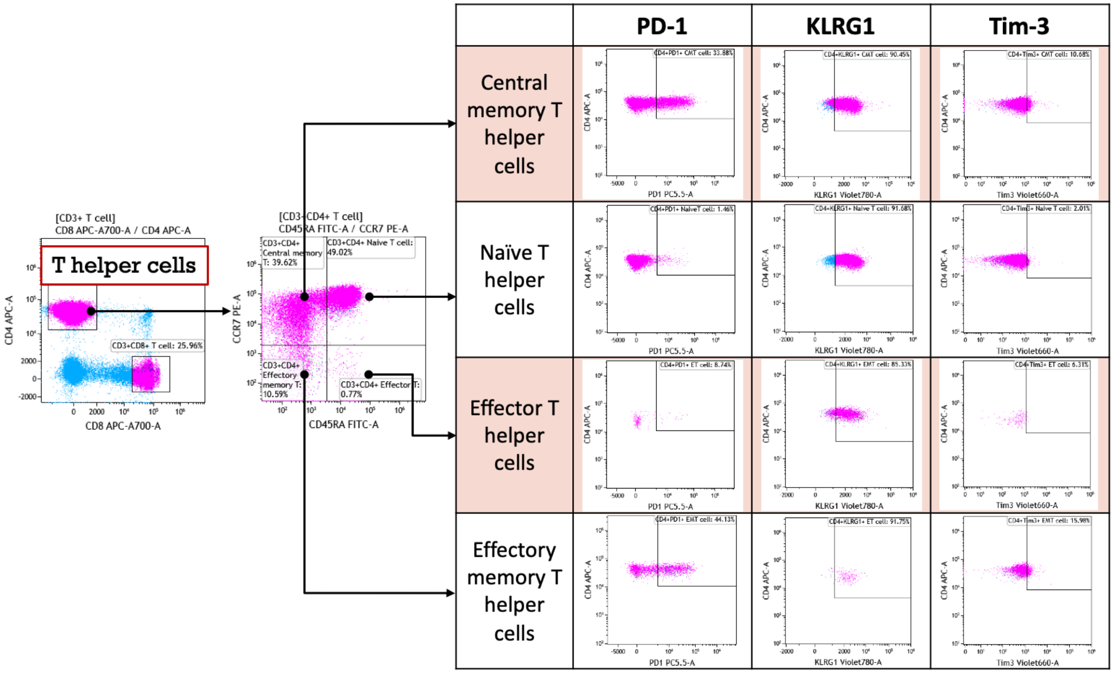
[CD3+ T cell]
CD8 APC-A700-A / CD4 APC-A
T helper cells
CD3+CD8+ T cell: 25.96%
CD4 APC-A
CD8 APC-A700-A
[CD3+CD4+ T cell]
CD45RA FITC-A / CCR7 PE-A
CD3+CD4+ Central memory T: 39.62%
CD3+CD4+ Naive T cell: 49.02%
CD3+CD4+ Effectory memory T: 10.59%
CD3+CD4+ Effector T: 0.77%
CCR7 PE-A
CD45RA FITC-A
PD-1
KLRG1
Tim-3
Central memory T helper cells
Naïve T helper cells
Effector T helper cells
Effectory memory T helper cells
PD1 PC5.5-A
KLRG1 Violet780-A
Tim3 Violet660-A

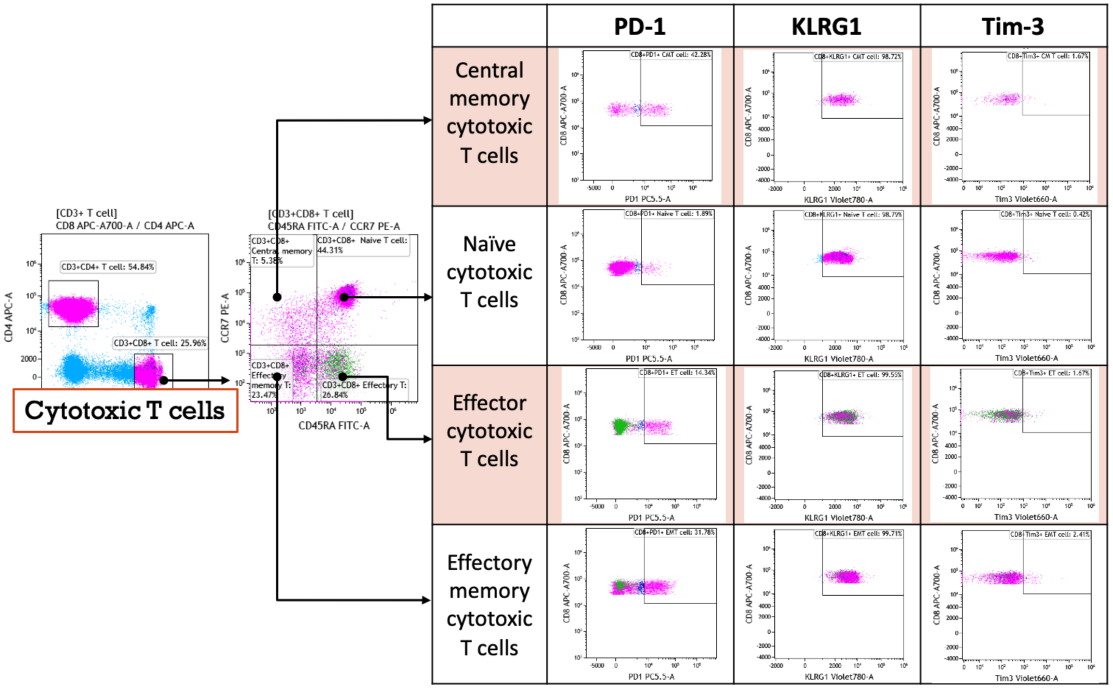

Cytotoxic T cells
[CD3+ T cell]
CD8 APC-A700-A / CD4 APC-A
CD3+CD4+ T cell: 54.84%
CD3+CD8+ T cell: 25.96%
CD4 APC-A
[CD3+CD8+ T cell]
CD45RA FITC-A / CCR7 PE-A
CD3+CD8+ Central memory T: 5.38%
CD3+CD8+ Naive T cell: 44.31%
CD3+CD8+ Effectory memory T: 23.47%
CD3+CD8+ Effectory T: 26.84%
CCR7 PE-A
CD45RA FITC-A
PD-1
KLRG1
Tim-3
Central memory cytotoxic T cells
Naïve cytotoxic T cells
Effector cytotoxic T cells
Effectory memory cytotoxic T cells
PD1 PC5.5-A
KLRG1 Violet780-A
Tim3 Violet660-A
CD8 APC-A700-A

Supplementary Figure 2. The cell gating of Treg cell immunophenotyping

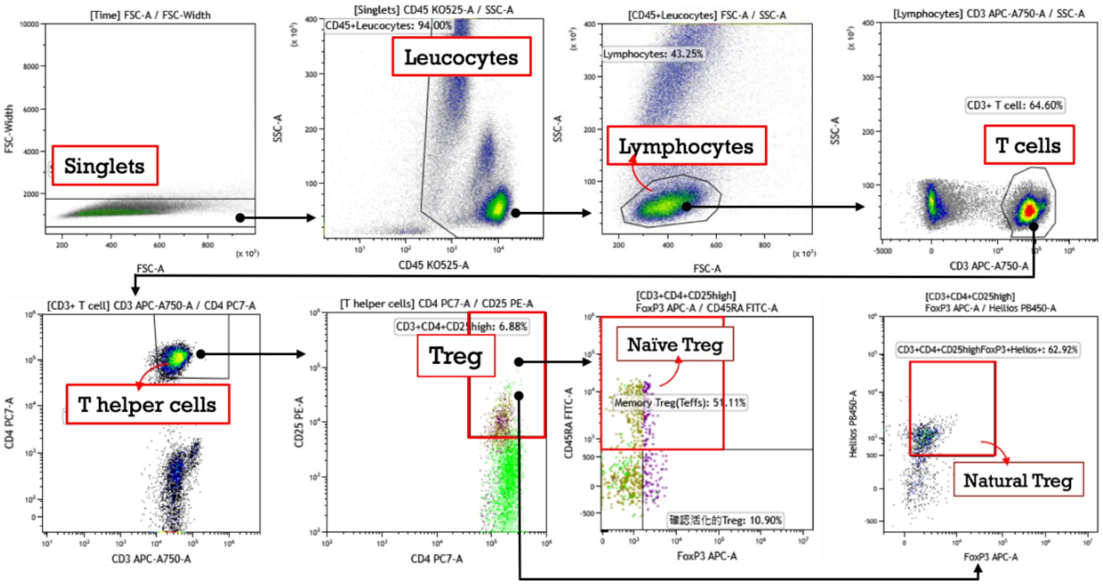

Supplementary Figure 3. The cell gating of B cell immunophenotyping

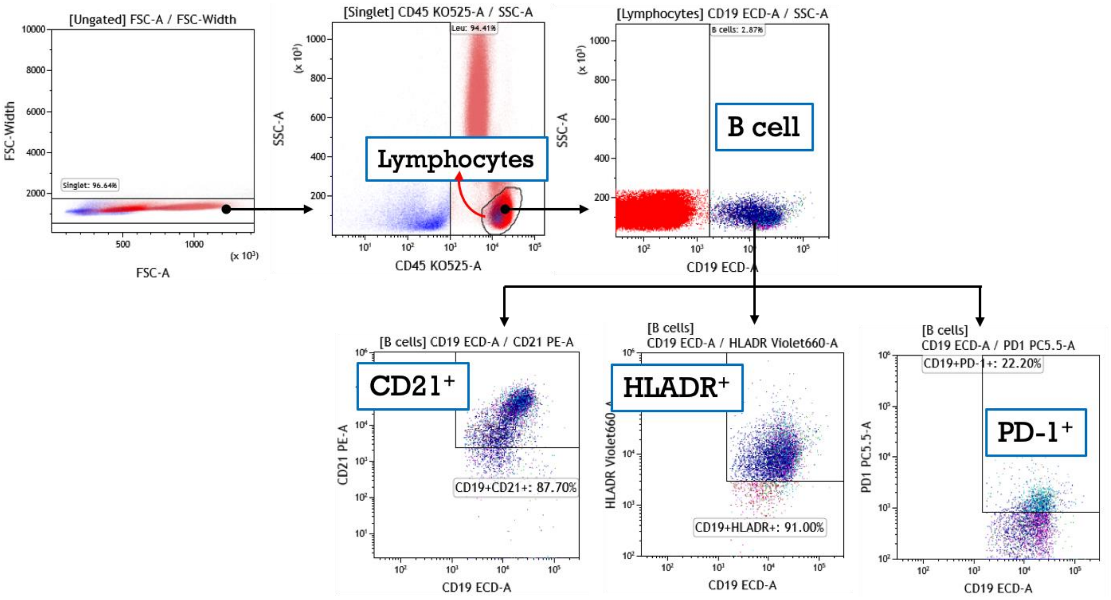

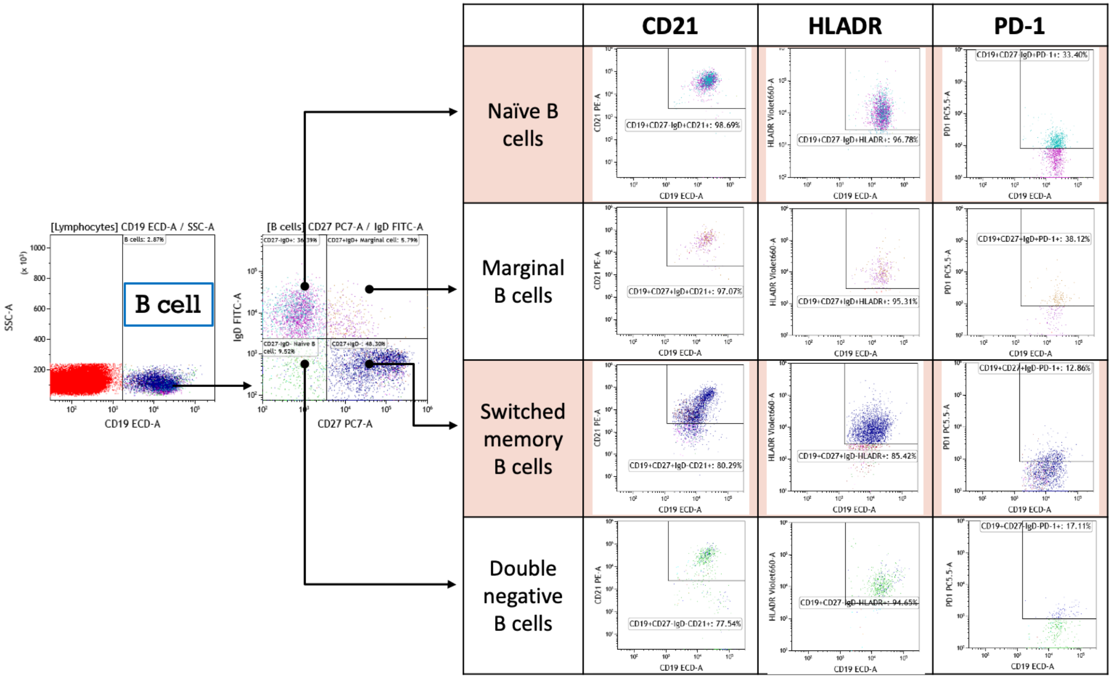
[Lymphocytes] CD19 ECD-A / SSC-A
B cell
SSC-A
CD19 ECD-A
[B cells] CD27 PC7-A / IgD FITC-A
IgD FITC-A
CD27 PC7-A
CD21
HLADR
PD-1
Naïve B cells
Marginal B cells
Switched memory B cells
Double negative B cells
CD19+CD27-IgD+CD21+: 98.69%
CD19+CD27-IgD+HLADR+: 96.78%
CD19+CD27-IgD+PD-1+: 33.40%
CD19+CD27+IgD+CD21+: 97.07%
CD19+CD27+IgD+HLADR+: 95.31%
CD19+CD27+IgD+PD-1+: 38.12%
CD19+CD27+IgD-CD21+: 80.29%
CD19+CD27+IgD-HLADR+: 85.42%
CD19+CD27+IgD-PD-1+: 12.86%
CD19+CD27-IgD-CD21+: 77.54%
CD19+CD27-IgD-HLADR+: 94.65%
CD19+CD27-IgD-PD-1+: 17.11%
CD19 ECD-A
